# Association between the Catechol-*O*-Methyltransferase (COMT) Val^158^Met Polymorphism and Manual Aiming Control in Healthy Subjects

**DOI:** 10.1371/journal.pone.0099698

**Published:** 2014-06-23

**Authors:** Guilherme M. Lage, Débora M. Miranda, Marco A. Romano-Silva, Simone B. Campos, Maicon R. Albuquerque, Humberto Corrêa, Leandro F. Malloy-Diniz

**Affiliations:** 1 Grupo de Estudo em Desenvolvimento Motor e Aprendizagem Motora (GEDAM), Departamento de Educação Física, Escola de Educação Física, Fisioterapia e Terapia Ocupacional, Universidade Federal de Minas Gerais, Belo Horizonte, Minas Gerais, Brazil; 2 Laboratório de Investigações em Neurociências Clínicas (LINC), Faculdade de Medicina, Universidade Federal de Minas Gerais, Belo Horizonte, Minas Gerais, Brazil; 3 INCT de Medicina Molecular, Faculdade de Medicina, Universidade Federal de Minas Gerais, Belo Horizonte, Minas Gerais, Brazil; 4 Departamento de Saúde Mental, Faculdade de Medicina, Universidade Federal de Minas Gerais, Belo Horizonte, Minas Gerais, Brazil; 5 Departamento de Pediatria, Faculdade de Medicina, Universidade Federal de Minas Gerais, Belo Horizonte, Minas Gerais, Brazil; 6 Departamento de Educação Física, Universidade Federal de Viçosa, Viçosa, Minas Gerais, Brazil; University Medical Center Goettingen, Germany

## Abstract

**Background:**

Prefrontal dopamine is catabolized by the catechol-*O*-methyltransferase (COMT) enzyme. Current evidence suggests that the val/met single nucleotide polymorphism in the COMT gene can predict the efficiency of executive cognition in humans. Individuals carrying the val allele perform more poorly because less synaptic dopamine is available.

**Methodology/Principal Findings:**

We investigated the influence of the COMT polymorphism on motor performance in a task that requires different executive functions. We administered a manual aiming motor task that was performed under four different conditions of execution by 111 healthy participants. Participants were grouped according to genotype (met/met, met/val, val/val), and the motor performance among groups was compared. Overall, the results indicate that met/met carriers presented lower levels of peak velocity during the movement trajectory than the val carriers, but met/met carriers displayed higher accuracy than the val carriers.

**Conclusions/Significance:**

This study found a significant association between the COMT polymorphism and manual aiming control. Few studies have investigated the genetics of motor control, and these findings indicate that individual differences in motor control require further investigation using genetic studies.

## Introduction

It is well known that dopaminergic functions play a critical role in human behavior and cognition. Several studies provide evidence indicating that dopaminergic neurotransmission in fronto-striatal circuits is related to complex cognitive processes, such as working memory [1], attention [2], decision-making [3] and inhibitory control [4]. The catechol-*O*-methyltransferase (COMT) enzyme is responsible for more than 60% of the metabolic degradation of dopamine in the frontal cortex [5]. Therefore, this enzyme has been associated with aspects of human cognition that are related to the dopaminergic system [6].

The COMT gene contains a functional polymorphism – va^l58^met – in which the enzyme activity is reduced by one third to one half in individuals homozygous for the val allele compared with the enzyme activity in individuals homozygous for the met allele [7]. Hence, the met allele results in a higher level of extrasynaptic dopamine than the val allele [8]. The heterozygote displays an intermediate enzyme activity [9].

Because of its crucial role in the prefrontal circuits, it has been hypothesized that these COMT polymorphism variants directly affect specific cognitive functions in humans. For example, studies suggest that individuals with the met allele present enhanced mental flexibility and capacity to shift [10], [11], [12] and working memory [13], [14], [15]. On the other hand, val/val homozygous allele was associated with better performance in a decision making task influenced by emotional processing [16].

Only few studies have focused their investigations on the relationship between COMT and motor control. Increasing evidence suggests that specific frontal lobe areas play a role in motor behavior [17] and that psychophysiological aspects involved in cognition interfere with motor control. The dorsolateral prefrontal cortex (DLPFC), a critical area for complex cognitive functions, has extensive interconnections with regions that are directly involved in motor functions, such as the premotor cortex and the supplementary motor area [18], [19]. Compared to the heterozygote and val homozygote, the met homozygote displays a stronger and more extended activation in the supplementary motor area, anterior cingulated cortex and DLPFC when performing response selection tasks (e.g., visual oddball task) [20].

When Bilder et al. [21] assessed the neurocognitive function in schizophrenic patients using a simple motor task (tapping finger task), they did not find an association between COMT and motor control. This finding may have emerged because the COMT polymorphism appears to be specifically associated with the performance of tasks that involve executive functions [22], such as tasks related to attentional, visuospatial and graphmotor skills [21]. The results found in a healthy sample by Walstrom et al. [23] reinforce this hypothesis. They found a significant association between COMT and motor performance in a task that places more demands on planning and monitoring, the Grooved Pegboard task, but they did not find significant association between COMT and motor performance in a tapping finger task. This type of repetitive task seems to be dependent on basal ganglia dopamine [23].

A good example of a motor task that requires executive functions is the goal-directed manual aiming movement [24]. Cognitive resources associated with the DLPFC are recruited during manual motor control, which enables us to hold information, to remember the desired goal, to resist distractions, to stay on task, to resist responding too early, and to inhibit a prepotent response [25]. All of these functions are related to motor response preparation and the monitoring of manual aiming movements [24]. Aiming movements performed with visual feedback consist of an initial impulse phase (a consequence of the response preparation) that roughly approaches the target by an open-loop control and a final homing component under a closed-loop control (related to the monitoring), with visually guided fine adjustments in the last phase of the movement [26].

We designed this study to investigate the association between the COMT val^158^met polymorphism and motor performance in an aiming task that has different sensory-motor requirements. Given that one of the key characteristics identified in the homozygous met allele carrier is enhanced performance during executive functions, we hypothesized that the met variant will be associated with better motor responses than the val variant in execution conditions that require inhibitory control, resistance to distraction and adaptation to perturbation in spatial accuracy. To our knowledge, no study investigating how the COMT polymorphism is specifically related to manual aiming control has been reported.

## Methods

### Participants

We studied 111 self-assigned Caucasian-Brazilians who ranged in age from 18 to 40 years old (64 women and47 men; mean age = 24.06±3.97). All of the participants were undergraduate students from local universities who were free of an Axis I diagnosis, as assessed by a psychiatrist using a structured interview (MINI-PLUS) and following the DSM IV criteria [27].

All of the participants were right-handed (handedness >.8 measured by the Edinburgh Handedness Inventory) [28] and had normal or corrected-to-normal visual acuity in both eyes. A local ethics committee approved all of the procedures, and the participants signed an informed consent sheet after receiving a full explanation of the study.

### Genotyping

Blood samples were collected only from health individuals who gave an informed consent to participate from the study. DNA samples were obtained from blood cells and extracted using the high salt method [29]. DNA samples were diluted in TE pH 8.0 and stored at −8°C. We perform amplification of DNA material by PCR-realtime and analyzed the COMT functional polymorphism (val^158^met- rs4680) using a TaqMan Genotype assay (Applied Biosystems, CA). PCR reaction was performed following the fabricant marker instructions and contained: 0.1 µl TaqMan Genotyping Master Mix, 3.4 µl deionizated water, 25 µg of DNA and 3.4 µl TaqMan Genotype Assay (Applied Biosystems, Foster City, CA). Reaction mix was prepared in a master mix pool and then distributed to each well to be mixture to the sample DNA. The PCR parameters included an initial denaturation at 95°C for 10 min followed by 50 cycles at 95°C for 15 seconds and 60°C for 1 minute. Genotype was determined based on the allelic discrimination mode (Strategene Mx3005 – MxPro QPCR- Software, 2007). Researchers involved in genotyping were blind to neuropsychological results and 10% of the genotypes were performed as a quality control.

### Apparatus

Apparatus were identical to that used by [30]. Manual aiming movements were quantified using a commercial digitizing tablet and the MovAlyzeR software. The sampling rate was 200 Hz. The tablet was attached to a MS Windows laptop computer running the MovAlyzeR software.

### Motor assessment

The motor task and procedures used by Lage et al. [30] were also used in this study. The protocol consisted of executing goal-directed manual movements using an inkless pen on a digitizing tablet. The participants were required to make rapid and accurate strokes with the pen from the home position to the target; these strokes were displayed in real time on the laptop monitor. A trial began by displaying both the home position and a filled-in green circle target to be pointed (the precue) on the monitor. The participant kept the cursor at the home position during this precue period. Next, the green target disappeared from the screen. The target then appeared as the imperative stimulus indicating “go” at random time intervals that ranged from 2 to 3 sec, and we began our recording during this phase of the trial. The participants were instructed to move the cursor to the target as quickly and accurately as possible. This procedure was our control condition and appeared in 70% of the trials.

Each of the other three conditions only appeared in 10% of the trials. Under the distractor condition, a filled-in yellow circle appeared instead of the green circle target (stimulus of the control condition). The goal under the distractor condition was identical to the control condition. The unique difference in this condition was the color of the stimulus. Under the inhibition of response condition, a filled-in red circle appeared, indicating “stop”, instead of the green circle target (stimulus of the control condition). Under this condition, the participant was instructed to not move the pen. The third condition was the higher index of difficulty condition. Under the higher index of difficulty condition, a filled-in green circle appeared that was similar to the target used in the control condition. The size and position of the green target, however, were different than the settings used under the control condition. The goal of executing the movement to the target as quickly and accurately as possible was still the same.

Under the control, distractor and inhibition of response conditions, the target (1 cm diameter) was presented at the same distance (19 cm center-to-center) and angle (45° upper right) from the home position, which resulted in an index of difficulty of 5.2 bits [31]. In the higher index of difficulty condition, the target displayed had a smaller diameter (0.5 cm), was further (20 cm center-to-center) from the home position, and was at a 35° upper right. The index of difficulty for this condition was 6.3 bits. A summary of all of the conditions is presented in Table 1.

**Table 1:** The characteristics of the all conditions of execution.

Condition	Target color	Target diameter	Target distance	Target angle	Goal	ID
Control	Green	1 cm	19 cm	45°	QA	5.2 bits
Distractor	Yellow	1 cm	19 cm	45°	QA	5.2 bits
IR	Red	1 cm	19 cm	45°	Stop	5.2 bits
HID	Green	0.5 cm	20 cm	35°	QA	6.3 bits

*Note:* IR = inhibition of response; HID = higher index of difficulty, FA = move as quickly and accurately as possible; ID = index of difficulty.

### Procedures

Prior to motor testing, the participants received standardized instructions concerning the general nature of the study. Participants held the inkless pen in a normal pen grip with their right hand. To get acquainted with the task and to find a comfortable posture, participants carried out six trials of the control condition (these trials were not analyzed). The body midline was aligned with the home position.

Immediately following familiarization, the motor task was performed. Participants performed 100 trials of the manual aiming task. The order in which conditions appeared was randomized in each block of 10 trials. Hence, in each block of 10 trials, there were 7 trials of the control condition and 1 trial of each one of the other conditions. This procedure was used to avoid that a specific condition would be concentrated more in a specific moment of the task.

After the presentation of the imperative stimulus, participants had 2 seconds to move from the home position to the target. After this interval, the target disappeared and the recording of the trial was finished. A red trace was displayed on the screen concomitantly with the movement to indicate the trajectory from the home position to the target. The entire test took approximately 16 minutes per participant to complete.

### Data reduction and dependent variables

Pen movements were low-pass filtered at 12 Hz using Fast Fourier Transform (FFT) and differentiated to yield estimates of the velocity and acceleration curves. A stroke was segmented into primary and secondary submovements by the first negative-to-positive zero crossing after the absolute peak velocity in the acceleration profile. The primary submovement refers to the initial part of the movement, the preprogrammed phase, and the secondary submovement refers to the online controlled phase.

The performance measures examined included the following: (1) reaction time, (2) movement time, (3) score of incorrect hits to the target (0 if hit and 1 if missed) and (4) score of response inhibition errors (0 if “stop” and 1 if “go”). The kinematic measures analyzed included the following: (1) peak velocity, (2) relative time to peak velocity and (3) number of discontinuities in acceleration in the secondary submovement (see details in Lage et al. [30]).

### Analysis

The mean values based on 10 trials for the distractor, inhibition of response and higher index of difficulty conditions were calculated for all dependent measures. For the control condition, the mean values based on 70 trials were calculated for all measures. The Kolmogorov-Smirnov Test revealed that the peak velocity violated the assumption of normal distribution under all test conditions, but the data were normalized by a logarithmic transformation (log10). Two-way analyses of variance (ANOVAs) were used to compare the genotype group performances (3 groups×3 conditions). The post-hoc analyses were performed with Duncan's new multiple range test. Chi-squared tests were used to analyze the following nominal data: (a) scores of incorrect hits to the target and (b) scores of response inhibition errors. The Bonferroni correction was used for multiple comparisons of the nominal data [32]. Significant difference at the level of .05 was adopted for all statistical analyses.

## Results

Sixty-four females (mean age 23.6±4.4) and 47 males (mean age 24.5±3.5) participated in this study. The genotype distribution was at Hardy-Weinberg equilibrium (x^2^ = 1.68; df = 1; *p* = 0.19). The number of male and female participants belonging to each genotype group is shown in Table 2.

**Table 2:** The total and relative number of female and male participants into each genotype group.

Gender	Genotype groups		
	MET/MET	MET/VAL	VAL/VAL
Female	14 (56%)	27 (56.3%)	23 (60.5%)
Male	11 (44%)	21 (43.7%)	15 (39.4%)

The analyses showed main effects of Groups Performance, *F*(2, 108) = 3.12, *p*<.05; Conditions, *F*(2, 216) = 27.23, *p*<.001, and interaction between Groups Performance and Conditions, *F*(4, 216) = 2.53, *p*<.05 to the measure of peak velocity. Post hoc comparisons revealed that the met/met allele group presented lower level of peak velocity under all of the conditions than the met/val allele group (*p*<.05). A marginal difference was also found between the met/met allele and val/val allele groups (*p* = .06), which indicates a tendency of lower level of peak velocity to the met/met allele group. The post hoc analysis on Conditions indicated higher level of peak velocity to the higher index of difficulty condition compared to the both control and distractor conditions (*p*<.001).

The main results found on the interaction between Groups Performance and Conditions were the following: (a) the met/met participants produced lower level of peak velocity under the control condition than in both the distractor and higher index of difficulty (*p*<.001); (b) the met/val and val/val participants produced lower level of peak velocity under the control condition than in the higher index of difficulty condition (*p*<.001), but there was no significant difference between control and distractor conditions (*p*>.05); (c) the met/met group presented lower level of peak velocity than the other groups under the control condition (*p*<.001); (d) the met/met group presented lower level of peak velocity than the met/val group under the distractor condition(*p*<.001), but there was no significant difference between met/met and val/val groups (*p*>.05); (e) the met/met group presented lower level of peak velocity than the other groups under the higher index of difficulty condition (*p*<.01).

The analyses of all the other measures related to the continuous data were not significant. The mean values and standard deviation of mean values of each measure for the genotype groups are shown in Table 3.

**Table 3:** Means and standard deviations of the genotype groups on dependent measures obtained in the control, distractor and higher index of difficulty conditions; results of the main effect of Groups performance (Anova) and Chi-squared test.

Measures	Conditions	Groups of genotype			*Value*	*p* value
		MET/MET	MET/VAL	VAL/VAL		
RT (sec)	CC	.40±.08	.41±.08	.41±.08	*F* = .01	.98
	CD	.40±.07	.40±.08	.40±.09		
	HIDC	.41±.08	.40±.07	.41±.09		
MT (sec)	CC	1.12±.18	.98±.26	1.07±.23	*F* = 2.78	.07
	CD	1.11±.19	.98±.26	1.06±.24		
	HIDC	1.11±.18	.98±.27	1.06±.25		
PV (cm/sec)	CC	26.78±7.15*	33.40±14.01*	28.45±10.20	*F* = 3.12	.05
	CD	24.54±7.26	33.52±14.61	28.11±10.07		
	HIDC	27.92±7.16	34.59±14.98	29.52±10.77		
RTPV (%)	CC	57.16±9.22	53.28±13.96	56.39±12.60	*F* = .78	.46
	CD	56.85±12.46	53.36±16.54	56.28±13.03		
	HIDC	56.92±11.93	53.33±15.56	55.72±14.83		
NDASS	CC	2.97±.87	2.58±.96	2.67±.89	*F* = 1.82	.16
	CD	3.03±1.02	2.55±1.09	2.62±.94		
	HIDC	3.02±1.13	2.49±1.18	2.77±1.19		
IH (%)	CC	17.42	25.55	22.10	*x^2^* = 28.87	.001
	CD	11.2	23.12	18.94	*x^2^* = 15.18	.01
	HIDC	33.6	38.54	43.68	*x^2^* = 6.58	.25

*Note:* RT = reaction time; MT = movement time; PV = peak velocity; RTPV = relative time to peak velocity; NDASS = number of discontinuities in acceleration in the secondary submovement; IH = score of incorrect hits to the target.

In the analyses of the score of incorrect hits to the target, the Chi-squared tests indicated the following results: (a) there was a significant difference among the groups under the control condition (*x^2^* = 28.87, p<.001); (b) there was a significant difference among the groups under the distractor condition (*x^2^* = 15.18, p<.01); (c) there was no significant difference among the groups under the higher index of difficulty condition (*x^2^* = 6.58, p = .25). For the multiple comparisons after the Bonferroni correction, a difference with a p value equal to or less than .01 was considered significant. Under the control condition, the results showed that the met/met allele group presented lower level of error of incorrect hits to the target than the met/val allele and val/val allele groups (p<.001, respectively). Furthermore, the val/val allele group exhibited lower level of error than the met/val allele group (p<.01). Under the distractor condition, the results showed that the met/met allele group presented lower level of error of incorrect hits to the target than the met/val allele and val/val allele groups (p<.01, respectively), and there was no significant difference between met/val allele and val/val allele groups (p = .03). The relative frequencies of the score of incorrect hits to the target are shown in Table 3.

In the analyses of the score of response inhibition errors, the Chi-squared test results indicate that there is no significant difference among the groups in the inhibition of response condition (*x^2^* = .04, p = .99). The frequency of errors resulting from response inhibition was 22.4% for the met/met allele group, 23.1% for the met/val allele group, and 22.8% for the val/val allele group.

## Discussion

We investigated the association between the COMT polymorphism and manual motor control based on the hypothesis that individuals homozygous for the met allele would have better motor responses than individuals carrying a val allele. Overall, this hypothesis was confirmed. The met/met allele group presented better spatial accuracy with the same movement time of the other groups. To our knowledge, this is the first study that investigates how the COMT polymorphism is specifically related to manual aiming control, and we found another important result, the val/met and val/val groups presented higher level of peak velocity than the met/met group.

A kinematic marker used to distinguish the preprogrammed phase from the visually guided phase of the movement is the peak velocity. The time interval preceding the peak velocity, the initial impulse phase, reflects the characteristics of the movement preparation. If we assume that frictional forces are negligible in our aiming task, then it is also possible to infer about peak force from peak velocity [30]. Curiously, individuals homozygous for the met allele exhibited lower level of peak velocity during their fast goal-directed movements than individuals carrying a val allele. It is widely accepted that in Parkinson disease the central dopamine deficiency impacts on peak muscle force [33], nevertheless, in our healthy and young sample, the better explanation for this difference in peak velocity among the groups seems to be related to aspects involved in executive functioning.

Dopamine plays an important role in all of the stages related to motor response preparation, such as when the performer's attention is focused on the relevant characteristics of the stimulus [34]. Met allele carriers exhibit a stronger and more extended activation during comparable task conditions, in which the activation of the supplementary motor area “…is related to ‘motivation’, respectively ‘task engagement’ and ‘selective attention’.” (Winterer et al., p. 1724 [20]). Our aiming task requires more from the motor system than the traditional key-press tasks but is similar in terms of its perceptual demands, which require a continuous comparison of the conditions. If the current evidence indicates that there is a significant association between the met allele and executive functioning, then why does the met homozygote produce lower level of peak velocity?

Another important variable that plays a role in the interference of fast goal-directed movements is spatial accuracy. It is possible that the met/met allele carriers executed more controlled movements and thereby generated a lower magnitude of peak force (peak velocity) during the trajectories. The main objective of this more controlled processing was to guarantee accuracy to the movement endpoint. Differences in the types of processing information appear to interfere with spatial accuracy. Explicit information processing, which is characterized by conscious, controlled and reflective processes, is productive in some contexts of execution. For example, low-impulsive subjects, in which explicit information processing predominates, exhibit a greater spatial accuracy than their more impulsive counterparts under the prepotent condition [30]. It is possible that the better attentional control observed in the met/met carriers in a key-press task [35] also improved the spatial accuracy of the met homozygote in our study.

Interestingly, the better spatial accuracy exhibited by the met/met allele carriers was not observed under the higher index of difficulty condition. The temporal and spatial demands on the motor system are greater under the higher index of difficulty condition than under the control and distractor conditions. It is possible that the executive advantage detected in the met allele homozygote overlaps with other aspects of motor execution. This result shows that the association between the COMT polymorphism and motor control depends on the sensory-motor aspects of the task. The higher index of difficulty condition presents a different size and position of the target, factors that require a more flexible motor control from the performer. In both healthy and psychiatric samples, individuals homozygous for the met allele show better performance in tasks requiring stable performance than individuals carrying a val allele. The same is not true in tasks requiring flexible performance [36], [37]. An increase in dopaminergic levels in the prefrontal cortex in tonic stages of neurotransmission is accompanied by a reduction of dopaminergic levels in subcortical structures during phasic transmission. This situation leads to less flexible responses and impaired modulation in response to environmental novelty [38]. The relationship between dopamine and cognitive functions is non-linear and inverted-U shaped, the response is optimized within a range of dopamine activity that is associated with the task demand [39], [40].

The met allele homozygote produced lower level of peak velocity under the control condition than under the distractor and higher index of difficulty conditions, but the val carriers under the control condition, presented lower level of peak velocity only when compared with peak velocity under the higher index of difficulty condition. Again, these findings seem to be related to inverted-U shaped relationship between dopamine and task demand. However, these differences in peak velocity under specific conditions do not reflect worse performance, but reveal different associations between the response preparation and the dopamine availability. The met/met allele group was more sensible to the changes of the conditions than the other groups. It reflected in alterations of the level of force produced during the movements when the conditions changed. This is the first study to investigate these types of associations. Further studies need to be conducted to analyze specific tasks and to investigate the proposal that the association between the COMT polymorphism and motor control depends on the sensory-motor aspects of the task. Furthermore, the relationship between the COMT polymorphism and the reaction and movement times should also be investigated. Is the COMT polymorphism associated with these measures during a motor task with no endpoint or a lower level of index of difficulty?

In conclusion, these findings indicate that there is a significant association between the COMT polymorphism and the manual aiming control in healthy subjects. This association was found to be specifically related to a component of movement preparation, the peak velocity. We did not find any association between the COMT polymorphism and online corrections during the monitoring phase of the movement. Furthermore, individuals homozygous for the met allele appear to be more accurate in the endpoint movement compared with the individuals carrying a val allele. It appears that individuals homozygous for the met allele were more efficient with an important aspect involved in a manual aiming task, the accuracy. Few studies have been reported that investigate functional polymorphisms and motor control in both clinical and non-clinical populations. For the first time, the influence of the COMT polymorphism on manual aiming control was observed and indicates that individual differences in motor control need to be investigated using genetic studies.
